# Racial and ethnic disparities in access to safe water and sanitation in high-income countries: a case study among the Arab-Bedouins of Southern Israel

**DOI:** 10.2166/washdev.2023.162

**Published:** 2023-08-21

**Authors:** Jesse D. Contreras, Haneen Shibli, Marisa C. Eisenberg, Ahmad S. Muhammad, Nadav Davidovitch, Mark A. Katz, Nihaya Daoud, Joseph N.S. Eisenberg

**Affiliations:** a Department of Epidemiology, University of Michigan, Ann Arbor, MI, United States; b School of Public Health, Faculty of Health Sciences, Ben-Gurion University of the Negev, Be’er Sheva, Israel; c PREPARED Center for Emergency Response Research, Be’er Sheva, Israel; d The Galilee Society for Health Research & Services, Rikaz Data Bank, Shefa-amer, Israel

**Keywords:** diarrheal disease, disparities, ethnic groups, sanitation, WASH, water

## Abstract

Disparities in access to water, sanitation, and hygiene within high-income countries are common and often occur across racial/ethnic lines. The Arab-Bedouins in Israel, a formerly nomadic ethno-national minority, have experienced displacement, forced sedentarization, and poverty since Israel was founded. Land disputes with the government have led to precarious living arrangements, including unrecognized villages that the government considers illegal. We administered a structured questionnaire in one government-planned, two legally recognized, and two unrecognized Bedouin communities in the Negev (190 households). Only 44% (95% CI 37%, 51%) of households had access to both safely managed drinking water and sanitation; nationally Israel reports over 99% coverage for each. In one unrecognized village, only 15% of households had access to safely managed water and sanitation, comparable to low-income countries. The overall 1-week prevalence of diarrhea in children under 5 years of age was 22% (95% CI 17%, 27%), with substantial variation between communities. These results highlight that universal access to safely managed drinking water and sanitation remains a relevant goal, not only for low- and middle-income countries but for high-income countries. Bedouin communities in the Negev are a prime example, emphasizing that historic gains in global development have not uniformly reached marginalized groups within high-income countries.

## INTRODUCTION

Access to safe drinking water and sanitation services are recognized as fundamental pillars of public health and have long been featured in global health and development agendas. Since global organizations first began monitoring access to safe drinking water and sanitation services, their attention has focused primarily on access in low- and middle-income countries. The World Health Organization (WHO) published the first report on the global status of urban drinking water in 1963, titled ‘Urban Water Supply Conditions and Needs in Seventy-Five Developing Countries’ and in 1975, published the first report on global excreta disposal titled ‘Community Water Supply and Excreta Disposal Situation in the Developing Countries: A Commentary.’ (Dieterich & Henderson 1963; Pineo & Subrahmanyam 1975; Bartram et al. 2014) These two reports included only those countries considered ‘developing’ by the United Nations (UN) at the time and were further limited to countries in Africa, Latin America, and Asia. Until 2000, global monitoring organizations continued to report access exclusively within four broad regions: Africa, Asia & Pacific, West Asia, and Latin America and Caribbean. (World Health Organization and UNICEF 1992, 1996; WHO and UNICEF 1993).

Today, increasing attention is being paid to inequalities in access to water, sanitation, and hygiene (WASH) within high-income countries, and targets of the Sustainable Development Goals (SDGs) call for achieving universal access to safe WASH services in all countries, regardless of income status (Winkler & Flowers 2017; University of North Carolina Water Institute 2020; Brooks et al. 2021; Mattos et al. 2021). In many cases, those facing WASH disparities within high-income countries are marginalized racial and ethnic groups, such as Black and Hispanic communities in the United States (U.S.) (Barton et al. 2015; Deitz & Meehan 2019; Flowers et al. 2019; Meehan et al. 2020), the Roma people in Europe (Anthonj et al. 2020), and Indigenous people across the world (Deitz & Meehan 2019; Meehan et al. 2020; Mattos et al. 2021). Migrant groups, refugees, and individuals experiencing homelessness, all of whom also are more likely to be racial or ethnic minorities, also face poor access to WASH services in high-income countries (Semenza et al. 2016; Capone et al. 2018; Dhesi et al. 2018; Frye et al. 2019; Tsesmelis et al. 2020). In this paper, we present a case study on WASH access among a minority ethnic group within a high-income country that faces health and socioeconomic inequalities related to political disputes, forced sedentarization, and legal battles over land rights: the Bedouins of the Negev region in Israel.

**Figure 1 ∣:**
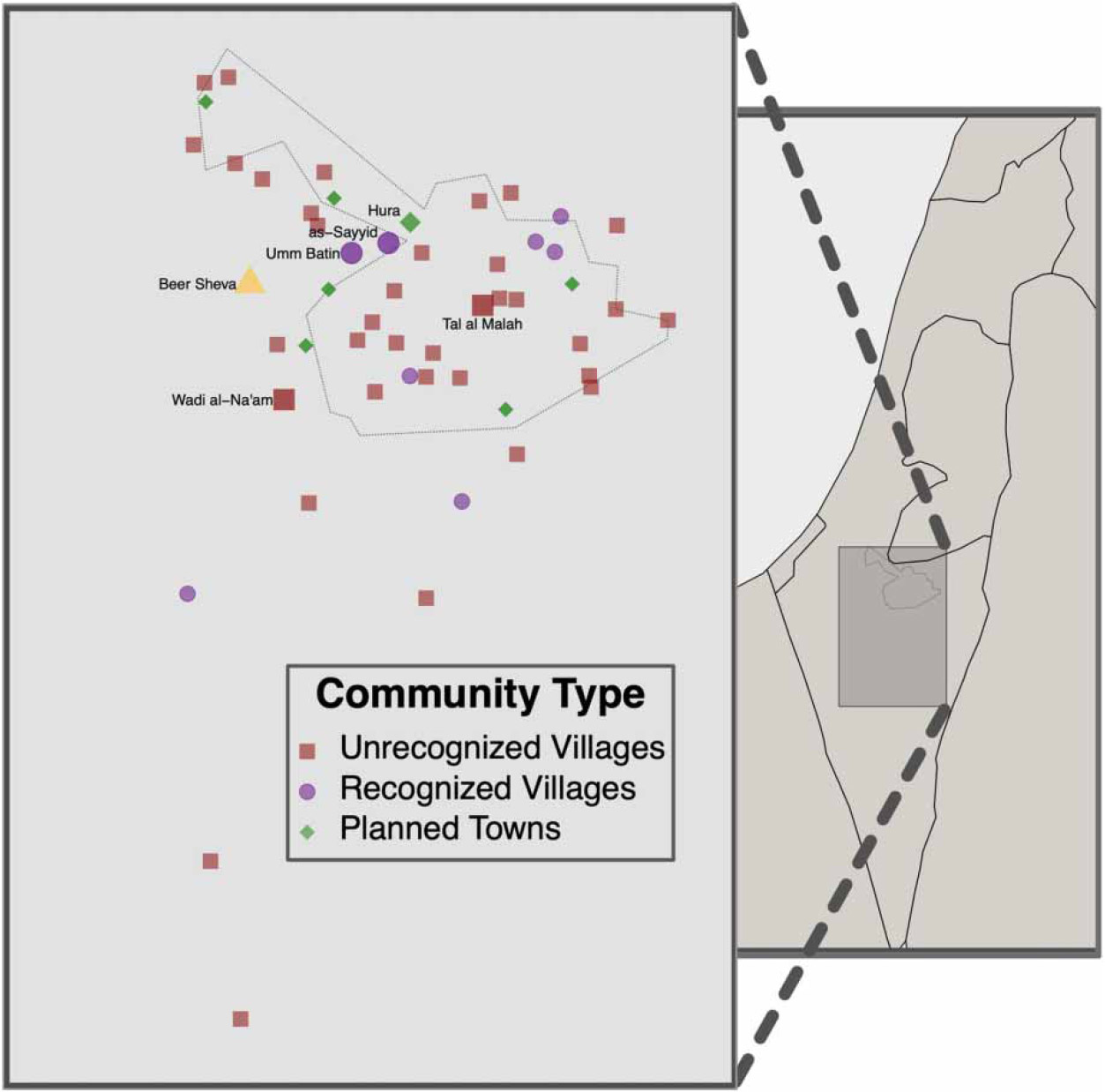
Bedouin villages in the Negev region of Israel, 2020. Bedouin villages include unrecognized villages (red squares), recognized villages (purple circles), and planned towns (green diamonds); the five communities included in our survey and Be’er Sheva are labeled by name. The approximate area of the Syag region is indicated with a dotted line.

The Arab-Bedouins (hereafter Bedouins) have lived throughout the Middle East and North Africa for centuries, including in the Naqab (Israel’s Negev Desert) since the 18th century. Following the Israeli War of Independence in 1948, the Israeli government confiscated large portions of Bedouin land (Kedar 2001). The government aimed to free that land for Jewish development, relocating many Bedouins to the smaller Syag region outside Be’er Sheva (Figure 1) (Yiftachel 1999; Shmueli & Khamaisi 2015). The Israeli government constructed seven planned towns for the Bedouins in the Syag between 1968 and 1989. An estimated 146,700 Bedouins (65%) in the Negev live in these planned towns today. Another 15,100 (7%) live on traditional tribal lands that have negotiated legal recognition by the Israeli government. An additional 55,700 (25%) live on traditional tribal lands that have not received formal recognition or legal status (Shmueli & Khamaisi 2015). These are known as unrecognized villages, although the government considers them ‘illegal’ villages. Construction or cultivation of cropland is frequently met with the threat or act of demolition. Bedouin villages, and unrecognized villages, in particular, have limited access to government services (Swirski & Hasson 2006; Rudnitzky & Abu Ras 2012). Bedouin people face the highest rates of poverty in Israel, and 80% of residents in unrecognized villages live in poverty (Abu-Bader & Gottlieb 2009). Bedouin adults face numerous physical and mental health disparities compared to other groups in Israel (Kordysh et al. 2005; Daoud et al. 2014; Al-Said et al. 2018). In addition, compared to the Jewish population in the Negev, Bedouin children have three times higher rates of infant mortality, higher proportions of underweight and vitamin deficiency, and increased hospitalization due to diarrhea (Benifla et al. 1997; Haas et al. 2014; Treister-Goltzman & Peleg 2015).

**Figure 2 ∣:**
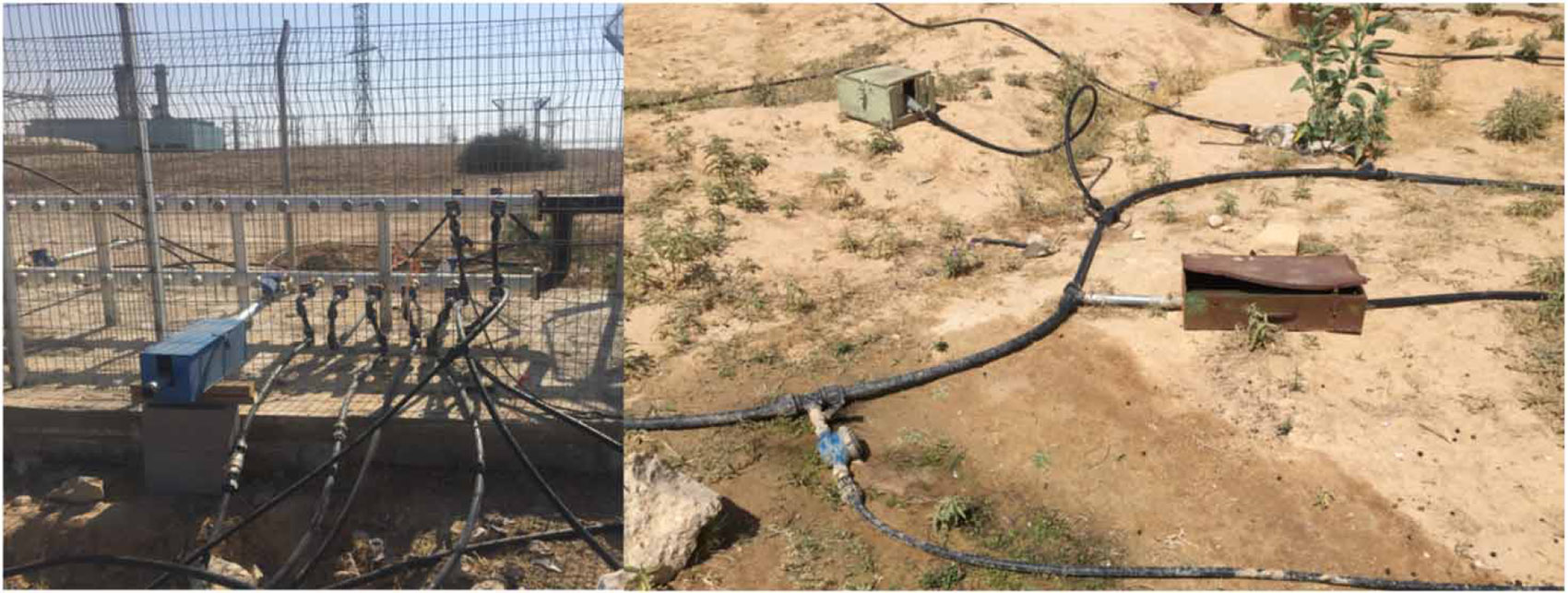
Examples of drinking water access for the Negev Bedouins. (Left) ‘Private’ standpipe allowing connections to the public water source; (Right) plastic hoses transporting water from a public source to Bedouin households. Printed with permission from Grace Christensen, Emory University.

The Negev Bedouins are legal citizens of Israel, including those living in unrecognized villages, and all Israeli citizens have a legal right to water (Murthy et al. 2013). However, easy accessibility is not a guaranteed right. To obtain water, households in unrecognized Bedouin villages can travel to pick-up locations or apply for private connections to a government standpipe (Figure 2) (Murthy et al. 2013). While the government may construct the connection from a public source to a Bedouin household located in an unrecognized village, it is not required to do so by law. The households are ultimately responsible for connecting their home to the pipe, and some Bedouins do so without obtaining legal permission. Unlike for drinking water, there is no legal right to sanitation services in Israel and thus the government is not required to provide sanitation services to Bedouins in unrecognized villages. However, even planned Bedouin towns, constructed by the government, have poorer sanitation services than in other areas of Israel (Shmueli & Khamaisi 2015). Poor access to sanitation in Bedouin communities possibly contributed to a 2013–2014 silent polio outbreak, in which no symptomatic cases were observed, that was identified from sewage samples in the planned town of Rahat (Brouwer et al. 2018).

To better understand WASH access among Israel’s Bedouin people, we measured access to drinking water and sanitation services through a household survey in five Bedouin communities in the Negev region. We also measured the prevalence of childhood diarrheal disease to estimate the impact of WASH on health in these communities. The survey was completed in one planned Bedouin town, two recognized villages, and two unrecognized villages to assess access to safe water and sanitation and child health among Bedouins of the Negev.

## METHODS

### Household sampling and survey methods

We conducted a cross-sectional survey in five Bedouin communities in the Negev with varying levels of formal government recognition: one planned town, two recognized villages, and two unrecognized villages. We aimed to enroll 200 households (40 from each community). Households were defined as people living in the same household unit and sharing food or expenses. Eligible households included at least one child under 5 years of age and one woman above 18 years of age willing to serve as the survey respondent. The Galilee Society, a national research non-profit focused on health among Arabs in Israel, selected communities for sampling that were previously open to working with outside organizations for research. The selected communities were Hura (a planned town with an estimated population of 18,800); Al-Sayyid (estimated population: 3,500); Umm Batin (estimated population: 4,000) (recognized villages); Wadi al-Na’am (estimated population: 9,000); and Tal al Malah (estimated population: 1,000) (unrecognized villages) (Figure 1). We conducted the survey between August 2019 and January 2020.

Interviewers were local Bedouin women who completed training by the research team. At the time we conducted our study, there were no available census maps of these Bedouin communities. We did not conduct a census due to the high likelihood that local communities would not agree to participate in a census, due to perceived implications regarding lands rights and occupancy. Thus, we employed a systematic sampling approach for door-to-door recruitment; for each of the five communities, interviewers chose a street and house at random to begin recruitment at the start of each day and moved along the street. Where houses were visible (indicating reasonable proximity and navigability) from one another and connected by walkable roads, interviewers skipped two houses between successful interviews. In more rural areas, where houses were not visible from one another or were not connected via walkable roads, interviewers sampled each eligible household encountered without skipping. The full survey in English is available in Supplementary Materials; the survey was translated into the local language (Arabic) in the United States and edited through consultations with national partners in Israel. The survey was further tailored to the local Bedouin context through testing with local Bedouin interviewers. Study activities were approved by Institutional Review Boards at the University of Michigan (HUM00130561) and Ben Gurion University of the Negev (2018–36).

### Study outcomes and socioeconomic measures

The primary outcomes measured were diarrheal disease in children under 5 years of age, access to safely managed drinking water, and access to safely managed sanitation. Diarrheal disease was measured for each child under 5 years of age in the household by asking respondents if the child had passed three or more loose or watery stools, or more loose or watery stools than usual, in a day during the last week. We determined the age of each child using the child’s reported birthdate and the date of survey completion. If only a birth year was provided, the midpoint of that year (July 1) was used for the birthdate. Children were excluded from child-level analyses if their birthdate-confirmed age was over 60 months or if the respondent did not provide a birthdate.

Safely managed drinking water is defined by the United Nation’s (UN) Joint Monitoring Program (JMP) as water from an improved source (piped water, boreholes or tubewells, protected dug wells, protected springs, rainwater, or packaged or delivered water) located on premises, available when needed, and free from fecal and chemical contamination (UNICEF (United Nation’s Children’s Fund) and WHO (World Health Organization) 2019). Based on three survey questions, we defined safely managed drinking water as water that (1) comes from an improved source, (2) is located in the home or yard, and (3) was available for at least part of each day in the last week. We considered piped water from the public supply as an improved source whether the connection was provided by the government, privately constructed with permission, or illegally constructed. We did not measure fecal or chemical contamination to fully measure the JMP’s definition of safely managed drinking water. Participants were also asked to describe water treatment strategies and disruptions in their water supply that lasted 24 h or more in the previous week. As one indicator of whether a household had access to safely managed water, we asked households to report on their main source of drinking water.

Safely managed sanitation is defined by the JMP as the use of improved facilities (flush and pour-flush toilets connecting to sewers, septic tanks or pit latrines, dry pit latrines with slabs, or composting toilets) that are not shared with other households and where excreta is safely disposed *in situ* or transported and treated off-site. (UNICEF (United Nation Children’s Fund) and WHO (World Health Organization 2019) Based on three survey questions, we defined safely managed sanitation as (1) the use of improved facilities, (2) where waste is not disposed of directly into the environment without collection, and (3) facilities are not shared with other households. We did not confirm the safe disposal of excreta beyond self-report by the household. Although households may have multiple sanitation facilities, we based indicators of safe management on the facility mainly used by household members for defecation.

Interviewers recorded the immunization status of children under five directly from vaccination booklets, if available, for fecal-oral viruses. The immunizations recorded were inactivated polio vaccine (IPV 1, IPV 2, and IPV 3), oral polio vaccine (bOPV 1 and bOPV 2), and rotavirus vaccine (rotavirus 1 and rotavirus 2). Indicator variables were created for children who were up to date on each immunization dose based on their age and national immunization recommendations (State of Israel Ministry of Health 2020). Numerous socioeconomic indicators were measured in the survey. Wealth was measured through ownership of various assets, which were adapted from previous DHS surveys in similar rural communities. An overall variable for household wealth was created by counting the number of assets owned by the household from a total of six: electricity, solar panels, refrigerator, air conditioner, washing machine, computer, and internet.

### Data analysis

We produced descriptive summaries of the count and prevalence or mean of each outcome and socioeconomic measure. We compared these values across all five communities to assess variability between communities and by type of village (planned town, recognized village, and unrecognized village) to examine the impact of legal status. Due to the limited sample size, we did not estimate statistical significance for differences in variables across villages or by village type. For our primary outcomes (diarrheal disease, safely managed drinking water, and safely managed sanitation), we calculated 95% confidence intervals around prevalence estimates to assess uncertainty. Confidence intervals were calculated as $p\pm 1.96*\sqrt{\frac{p*(1-p)}{n}}$, where p is the prevalence estimate, and n is the sample size. Data analysis was completed using R software.

## RESULTS

### Socioeconomic characteristics

We sampled between 33 and 40 households from each Bedouin village, resulting in a total of 190 household surveys completed (Table 1). Overall, SES indicators were highest in Hura (a planned town), Al-Sayyid (a recognized village), and Tal al Malah (an unrecognized village). Women from Tal al Malah in our sample had the highest education; 55% had completed university or technical college, and 53% were currently employed. Households from Wadi al-Na’am, the other unrecognized village, had the lowest SES overall.

### Household WASH access

In total, 57% (95% CI 50%, 64%) of households had access to safely managed drinking water services (75% in Hura, 51% in Al-Sayyid, 61% in Umm Batin, 71% in Wadi al-Na’am, and 24% in Tal al Malah). Most households (97%) reported that their main source of drinking water was piped to their home or yard (Table 1). However, only half of the households were provided a connection to piped water by the government. Furthermore, just 5% of households in Wadi al-Na’am and 30% of households in Tal al Malah, the two unrecognized villages, had government-supplied piped water. Most other households, mostly in the unrecognized villages, reported piped drinking water that was not provided by the government, i.e., the household constructed their own piping to connect their household to a government water line. Intermittency of water supply was high, as 41% of households reported that their main drinking water source had been unavailable for at least one full day in the past week, including almost three-quarters of households in Tal al Malah. On average, the planned town had the highest access to safely managed water (75%), followed by recognized villages (53%) and unrecognized villages (45%).

Overall, 63% (95% CI 56%, 70%) of households had access to sanitation facilities that met our definition of safely managed (75% in Hura, 86% in Al-Sayyid, 50% in Umm Batin, 88% in Wadi al-Na’am, and 15% in Tal al Malah). All households in Hura, Al-Sayyid, Umm Batin, and Tal al Malah and two-thirds of households in Wadi al-Na’am reported a flush toilet or pour-flush latrine as their main sanitation facility (Table 1). The remaining households in Wadi al-Na’am reported access to a pit latrine. Despite reporting high access to flush toilets, only 39% of households in the total sample with a flush toilet or latrine reported that their flushed waste went to a piped sewer system (75% in Hura, 41% in Al-Sayyid, 30% in Umm Batin, zero in Wadi al-Na’am, and 23% in Tal al Malah). In 30% of households with a flush facility, sewage was flushed directly into the environment outside or near the home (23% in Hura, zero in Al-Sayyid, 42% in Umm Batin, 4% in Wadi al-Na’am, and 78% in Tal al Malah). The remaining households flushed their waste into a pit latrine, septic tank, or cesspit. The planned town had the highest access to safely managed sanitation (75%) on average, followed by recognized villages (69%) and unrecognized villages (51%).

In total, 44% (95% CI 37%, 51%) of households had access to both a safely managed drinking water source and safely managed sanitation (60% in Hura, 43% in Al-Sayyid, 35% in Umm Batin, 63% in Wadi al-Na’am, and 15% in Tal al Malah). Access to both water and sanitation was highest on average in the planned town (60%), followed by unrecognized villages (38%) and recognized villages (37%).

### Children’s health

Across all five communities, there were 264 children under 5 years of age confirmed by their birthdate from 168 households (Table 2). Caregivers reported that 57 of those children had diarrhea in the previous week, resulting in an overall prevalence of 22% (95% CI 17%, 27%). The prevalence of caregiver-reported diarrhea was highly variable by village including zero cases in Al-Sayyid, 9% of children in Hura, 68% in Umm Batin, 18% in Wadi al-Na’am, and 24% of children in Tal al Malah. The average diarrheal prevalence was highest in recognized villages (32%), followed by unrecognized villages (20%), and the planned town (9%).

Coverage with immunizations against polio was higher than against rotavirus (Table 2). Among children old enough for the vaccine, 92% received the first dose of IPV and 83% received the third dose. Eighty-four percent of eligible children received each dose of OPV. In contrast, only 61 and 56% of eligible children received the first two doses of rotavirus vaccine, respectively. Immunization coverage varied substantially between communities but was lowest in Wadi al-Na’am. No children in Wadi al-Na’am had received any dose of rotavirus vaccine and only 13% of eligible children had received the second or third dose of IPV.

## DISCUSSION

### Household WASH access

Only 44% of households in our sample had access to both a safely managed drinking water source and safely managed sanitation facilities. While nearly all households in our sample had access to piped drinking water, the government did not provide connections for about half of all households, and for three-quarters of households in unrecognized villages. The remaining households constructed their own connection to the public water source. We did not ask households to report the legality of their connection, but the potential dependency of some households on illegal connections suggests that the presence of piped water is not necessarily a secure source. The government has the legal authority to remove an illegal connection at any time. This action would not violate the government’s responsibility to supply drinking water to all Israeli citizens, as the presence of ‘water centers’ near recognized villages is legally considered sufficient access for Bedouins living in unrecognized villages despite being located many kilometers from the water center (Murthy et al. 2013). The precarious nature of illegal connections for these households highlights the role of water governance and social power in water security (Bakker & Morinville 2013). Separate from physical or financial limitations to water security that is commonly observed in lower-income countries, governmental and social factors produce stark disparities in water security even within high-income countries such as Israel.

Another challenge facing Bedouins was the availability of piped water when it is needed. Almost half of all households reported that their main drinking water source had been unavailable for 24 h or more during the last week, possibly due to low pressure in the plastic hoses that transport water to Bedouin households over large distances. The water that arrives is not immediately drinkable. The plastic hoses sit on top of the desert sand and are directly exposed to the sun, which results in extremely hot water arriving at households (Figure 2). Especially for households without refrigeration, which includes 47% of our sample in Wadi al-Na’am, this water is not available for use when needed. The definition of safely managed drinking water includes availability when needed due to the social and health consequences of intermittent supply (WHO (World Health Organization) 2017). Households with intermittent supplies face adverse health outcomes due to compromised water quality, recontamination of water that is stored for later use, and restriction of intake when water is unavailable (Cifuentes et al. 2002; Abu Amr & Yassin 2008; Levy et al. 2008; Ercumen et al. 2014; Kumpel & Nelson 2016; WHO (World Health Organization) 2017).

Use of non-flushing latrines was only reported in the unrecognized village of Wadi al-Na’am. We considered improved latrines as safely managed by design; we did not collect additional details that would have allowed us to characterize the extent to which improved latrines were part of a safely managed sanitation system, such as practices for managing a full pit. The full extent of safe management for latrines depends on the presence of adequate services and resources for pit emptying or covering and replacing. All other households in Wadi al-Na’am reported using a toilet or latrine that flushes. However, many households reported that their flushed waste went directly into the environment, which we did not define as safely managed. Waste is flushed directly outside of the home for some households, creating a direct point of exposure to enteric pathogens. For other households, waste is flushed to nearby wadis (dry streambeds that fill during winter), which can result in groundwater contamination (Alhamid et al. 2007; Klawitter et al. 2007; Daraowsheh 2014). In addition, the presence of fecal waste in dry wadis enables potential human contact with contaminants, such as through children playing near wadis or spread by animals and flies (Baker et al. 2018).

Notably, our sample of households in Tal al Malah had the worst access to water and sanitation despite their relatively high SES. The vast majority of households sampled in Tal al Malah lacked access to safely managed drinking water and safely managed sanitation services. These results suggest that even wealthier, highly educated households within some unrecognized villages are not able to access safe WASH services and that the legal status of Bedouin villages is a substantial barrier to achieving access.

### Children’s health

The prevalence of diarrheal disease among Bedouin children in our sample was high (22% overall). Coverage with immunizations against fecal-borne pathogens was mixed but substantially lower than for other populations in Israel (World Health Organization and UNICEF 2019). Coverage was uneven between villages, as few children received these immunizations in Tal al Malah, which possibly explains a portion of the high diarrheal burden among Bedouin children. The large proportion of households depending on intermittent water supplies and with poor or no fecal waste management might further explain this high morbidity.

Poor WASH access among Bedouin communities also might help to understand a 2013–2014 silent outbreak of poliovirus (in which no symptomatic cases were observed) that occurred in Israel, with sustained transmission in Rahat and the surrounding region (Brouwer et al. 2018). The results of our survey in Hura demonstrate that, even in planned Bedouin towns, households struggle with intermittent drinking water supply and dump sewage into the environment without collection. The potential for fecal-borne diseases to spread between Bedouin communities and into other regions of Israel remains high due to poor WASH access, and that risk potentially is exacerbated by the legal status of unrecognized Bedouin villages.

There are three main limitations of our study. First, the sample size within each of the five communities was small, which limits comparability between communities. Second, the lack of a representative sample may have resulted in unaccountable sampling bias. Third, we relied on self- or caregiver-reported outcomes, which are open to measurement bias, particularly between communities. Together, these limitations might explain the high degree of variability in diarrheal prevalence that we estimated between communities. The diarrheal prevalence measured in Al-Sayyid (0%) and Umm Batin (68%) stands out as extreme estimates within our sampled communities. The small sample size within a given community might have resulted in these extreme estimates by chance, for example, if healthy children from Al-Sayyid and infected children from Umm Batin were oversampled by chance (or due to an outbreak, in the case of Umm Batin). Variability also could reflect reporting bias due to differential understanding of the question by community; additionally, some participants may have reported what they thought the interviewer wanted to hear (e.g., social desirability or courtesy bias). Although the extreme estimates measured for diarrheal disease stand out, these same limitations apply to estimates for all outcomes.

### Disparities in high-income countries

Our results underscore that global health inequities are not limited to those between higher- and lower-income countries. Israel is a high-income country, with a gross domestic product (GDP) per capita that is just above that of France and Japan (The World Bank 2020). Despite the high economic production of Israel overall, less than half of Bedouin households in our study had access to both safely managed drinking water and sanitation services. In contrast, over 99% of the overall population in Israel was estimated to have access to safely managed drinking water in 2017, and 94% had access to safely managed sanitation services (UNICEF (United Nation’s Children’s Fund) and WHO (World Health Organization) 2019). We measured those same coverage levels in Tal al Malah as 24 and 15%, respectively. Access in Tal al Malah was even lower than for the 47 ‘least developed countries’, as defined by the UN, where 35% of households had access to safely managed drinking water in 2017 and 34% had access to basic sanitation services (UNICEF (United Nation’s Children’s Fund) and WHO (World Health Organization) 2019; United Nations Department of Economic and Social Affairs 2020). Likewise, the prevalence of diarrheal disease we estimated in two unrecognized villages combined (20%) is about the same as that measured by Demographic and Health Surveys in Burkina Faso (1992 & 1999), Ghana (1993 & 2008), Cambodia (2005), Bolivia (1998), and Nigeria (2003), among others (Fuller et al. 2015). In contrast, the combined average incidence of diarrhea among children under five in high-income countries was just 1% per week in 2016 (Troeger et al. 2018).

The Bedouin of Israel are an example of stark disparities in health and WASH access that persist within high-income countries across the world, often across racial and ethnic lines. In rural areas in the U.S. where municipal sanitation systems are not available, many people, especially in predominantly Black communities, have limited resources to construct on-site systems and often resort to directly piping sewage from the home and into yards (Flowers et al. 2019). Unlike in unrecognized Bedouin villages, where construction is actively blocked or met with demolition by the national government over land rights (Murthy et al. 2013), poor sanitation access for these rural households in the U.S. often results from a lack of funding for non-municipal sanitation (Flowers et al. 2019). These coverage issues often are not captured by national WASH metrics, which still report nearly universal access to safely managed services in the U.S. and other high-income countries (Meehan et al. 2020).

The Bedouins of Israel share characteristics with other minority groups within high-income countries that face limited WASH access. Many Roma people throughout Europe live in informal settlements or as seminomads, which contribute to substantial WASH disparities faced by Roma populations (Anthonj et al. 2020). Poor WASH access in Hispanic colonies along the U.S.–Mexico border is associated with limited access to the formal economy (Barton et al. 2015). And like the Bedouins, a history of forced migration from traditional lands, land seizure, and economic marginalization through settler colonialism has led to longstanding WASH challenges for Indigenous peoples throughout the world (Meehan et al. 2020; Mattos et al. 2021; Hartwig et al. 2022).

## CONCLUSION

Historically, the challenges that marginalized populations in wealthy countries face in accessing safely managed drinking water and sanitation services are left out of national statistics, including decades of global WASH monitoring that excluded high-income countries until the year 2000. As a result, public perception of WASH access in high-income countries has generally reflected access among groups in power (Meehan et al. 2020). As more attention is paid to inadequate WASH access among marginalized populations, our study highlights the importance of considering the cultural and political challenges that engender these disparities. Common barriers that impede progress toward access to safely managed water and sanitation, including budgetary, management, and legal, should be analyzed through a lens of political economy with special attention to groups with limited political power. Any sustainable intervention should be informed by a thorough understanding of these barriers and how to overcome them within the local social and political context. In the spirit of the Sustainable Development Goal of universal access of safely managed water and sanitation, the JMP should advocate for countries to provide disaggregated information across marginalized subpopulations to track progress toward ending these disparities.

## Supplementary Material

Supplementary Material

## Figures and Tables

**Table 1 ∣:** Socioeconomic characteristics and WASH access among Bedouin households

	Planned town Hura	Recognized villages			Unrecognized villages			Total –
		Al-Sayyid	Umm Batin	Total	Wadi al-Na’am	Tal al Malah	Total	
Households surveyed, *N*	40	37	33	70	40	40	80	190
Range of dates for survey completion	Nov 14, 2019–Jan 3, 2020	Jul 24–Dec 10, 2019	Aug 24–Dec 28, 2019	Jul 24–Dec 28, 2019	Jul 24–Jul 25, 2019	Aug 18–Oct 3, 2019	Jul 24–Oct 3, 2019	Jul 24, 2019 - Jan 3, 2020
*Demographics and* *socioeconomics*								
Highest level of education completed by respondent:								
University or technical college, *N* (%)	22 (55)	6 (16)	9 (27)	15 (21)	3 (8)	22 (55)	25 (31)	62 (33)
High school, *N* (%)	11 (28)	22 (59)	20 (61)	42 (60)	11 (28)	10 (25)	21 (26)	74 (39)
Less than high school, *N* (%)	7 (18)	9 (24)	4 (12)	13 (19)	26 (65)	8 (20)	34 (43)	54 (28)
Respondent is employed, *N* (%)	11 (28)	7 (19)	7 (21)	14 (20)	8 (21)	21 (53)	29 (36)	54 (29)
Household owns livestock, N (%)	9 (23)	7 (19)	12 (36)	19 (27)	9 (23)	10 (25)	19 (24)	47 (25)
Husband has more than one wife, *N* (%)	8 (21)	6 (17)	7 (24)	13 (19)	14 (36)	10 (26)	24 (30)	45 (25)
Female respondent required to be accompanied to go to the doctor, *N* (%)	12 (30)	5 (14)	14 (42)	19 (27)	14 (35)	33 (83)	47 (59)	78 (41)
Number of household assets out of six^a^: mean (SD)	3.9 (1.7)	4.1 (1.2)	2.6 (1.6)	3.4 (1.6)	1.2 (1.0)	2.6 (1.6)	1.9 (1.5)	2.9 (1.8)
*Water and sanitation* *access*								
Drinking water								
Piped to home or yard provided by government, *N* (%)	31 (78)	35 (95)	16 (48)	51 (73)	2 (5)	12 (30)	17 (21)	96 (51)
Piped to home or yard; not provided by government, *N* (%)	9 (23)	1 (3)	16 (48)	17 (24)	33 (83)	28 (70)	61 (76)	87 (46)
Collected from public source, *N* (%)	0	0	0	0	5 (13)	0	5 (6)	5 (3)
From well, *N* (%)	0	1 (3)	1 (3)	2 (3)	0	0	0	2 (1)
Drinking water unavailable from main source for ≥ 24 h in last week, *N* (%)	10 (25)	17 (46)	12 (36)	29 (41)	9 (23)	29 (73)	38 (48)	77 (41)
Makes drinking water safer, e.g., boils water, *N* (%)	22 (55)	7 (19)	11 (33)	18 (26)	2 (5)	37 (93)	39 (49)	79 (42)
Number of households with safely managed drinking water source, *N*	30	18	19	37 (53)	27	9	36 (45)	103
Proportion of households with safely managed drinking water source, % (95% CI)	75 (62, 88)	51 (35, 67)	61 (44, 78)	53 (41, 65)	71 (57, 85)	24 (11, 37)	45 (34, 56)	57 (50, 64)
Sanitation facility:								
Toilet that flushes or a pour-flush latrine, *N* (%)	40 (100)	37 (100)	33 (100)	70 (100)	27 (68)	40 (100)	67 (84)	177 (93)
Ventilated improved pit latrine, *N* (%)	0	0	0	0	1 (3)	0	1 (1)	1 (1)
Pit latrine with slab, *N* (%)	0	0	0	0	11 (28)	0	11 (14)	11 (6)
Pit latrine without slab, *N* (%)	0	0	0	0	1 (3)	0	1 (1)	1 (1)
Shared sanitation facility between two or more households, *N* (%)	1 (3)	4 (11)	4 (13)	8 (11)	2 (5)	4 (10)	6 (8)	15 (8)
Sewage goes to:								
Piped sewer system (if flush toilet/latrine), *N* (%)	30 (75)	15 (41)	10 (30)	25 (36)	0	9 (23)	9 (13)	64 (36)
Septic tank or cesspit (if flush toilet/latrine), *N* (%)	1 (3)	3 (8)	4 (12)	7 (10)	0	0	0	8 (5)
Pit latrine (if flush toilet/latrine), *N* (%)	0	19 (51)	5 (15)	24 (34)	25 (93)	0	25 (37)	49 (28)
Environment (if flush toilet/latrine), e.g., yard or ravine, *N* (%)	9 (23)	0	14 (42)	14 (20)	1 (4)	31 (78)	32 (48)	55 (31)
Households with safely managed sanitation facilities, *N*	30	32	16	48 (69)	35	6	41 (51)	119
Proportion of households with safely managed sanitation facility, % (95% CI)	75 (62, 88)	86 (75, 97)	50 (33, 67)	69 (58, 80)	88 (78, 98)	15 (4, 26)	51 (40, 62)	63 (56, 70)
Households with safely managed drinking water and safely managed sanitation, *N*	24	15	11	26 (37)	24	6	30 (38)	80
Proportion of households with safely managed drinking water and safely managed sanitation, % (95% CI)	60 (45, 75)	43 (27, 59)	35 (19, 51)	37 (26, 48)	63 (48, 78)	15 (4, 26)	38 (27, 49)	44 (37, 51)
Interviewer observed soap available at the main handwashing location if permitted access, *N* (%)	31 (78)	30 (97)	18 (62)	48 (80)	23 (62)	20 (65)	43 (63)	122 (73)

a Electricity, refrigerator, air conditioner, washing machine, computer, and internet.

**Table 2 ∣:** Health and immunization status of children under 5 years old in sampled Bedouin households

	Planned Town Hura	Recognized Villages			Unrecognized Villages			Total –
		Al-Sayyid	Umm Batin	Total	Wadi al-Na’am	Tal al Malah	Total	
Children under five confirmed by birthdate, *N*	56	46	41	87	58	63	121	264
Households with at least one child confirmed under five years, *N* (%)	37 (93)	27 (73)	30 (91)	57 (81)	34 (85)	40 (100)	74 (93)	168 (88)
Average number of children > 5 per household, mean	1.5	1.7	1.4	1.5	1.7	1.6	1.6	1.6
Children with diarrhea in last week, *N*	5	0	28	28	9	15	24	57
Proportion of children with diarrhea in last week, % (95% CI)	9 (2, 16)	–	68 (54, 82)	32 (22, 42)	18 (8, 28)	24 (13, 35)	20 (13, 27)	22 (17, 27)
Child With Diarrhea Given:								
More to drink than usual, *N* (%)	3 (60)	–	9 (32)	9 (32)	3 (33)	3 (20)	6 (25)	18 (32)
Less to drink than usual, *N* (%)	1 (20)	–	11 (39)	11 (39)	0	3 (20)	3 (13)	15 (26)
More to eat than usual, *N* (%)	0	–	5 (18)	5 (18)	0	1 (7)	1 (4)	6 (11)
Less to eat than usual, *N* (%)	3 (60)	–	18 (64)	18 (64)	1 (11)	0	1 (4)	22 (39)
Healthcare sought for child’s case of diarrhea, *N* (%)	4 (80)	–	5 (18)	5 (18)	4 (44)	9 (60)	13 (54)	22 (39)
Child received oral rehydration liquid or packet for diarrhea case, *N* (%)	2 (40)	–	1 (4)	1 (4)	0	7 (47)	7 (29)	10 (18)
Child received antibiotic pill or syrup for diarrhea case, *N* (%)	1 (20)	–	4 (14)	4 (14)	0	1 (7)	1 (4)	6 (11)
Children with fever in last week, *N* (%)	7 (13)	2 (4)	8 (20)	10 (11)	6 (10)	9 (14)	15 (12)	32 (12)
Eligible Children Documented Receipt Of:								
First dose of Rotavirus vaccine^a^, *N* (%)	23 (59)	15 (60)	35 (90)	50 (78)	0	34 (67)	34 (46)	111 (61)
Second dose of Rotavirus vaccine, *N* (%)	20 (51)	15 (65)	28 (72)	43 (69)	0	34 (68)	34 (47)	97 (56)
First dose of inactivated polio vaccine (IPV 1)^b^, *N* (%)	38 (97)	22 (88)	38 (97)	60 (90)	13 (57)	51 (100)	64 (86)	162 (92)
Second dose of inactivated polio vaccine (IPV 2), *N* (%)	38 (97)	20 (87)	38 (97)	58 (94)	3 (13)	50 (100)	53 (73)	149 (86)
Third dose of inactivated polio vaccine (IPV 3), *N* (%)	34 (92)	17 (77)	37 (97)	54 (90)	3 (13)	46 (100)	49 (71)	137 (83)
First dose of oral polio vaccine (OPV 1)^c^, *N* (%)	34 (92)	17 (77)	29 (76)	46 (77)	17 (74)	42 (91)	59 (86)	139 (84)
Second dose of oral polio vaccine (OPV 2), *N* (%)	30 (97)	14 (78)	28 (85)	42 (82)	8 (42)	36 (97)	44 (79)	116 (84)

a Rotavirus schedule: first dose at 2 months, second dose at 4 months, third dose at 6 months (not included in our survey).

b IPV schedule: first dose at 2 months, second dose at 4 months, third dose at 6 months.

c OPV schedule: first dose at 6 months, second dose at 18 months (recommended that children receive both IPV and OPV).

## Data Availability

Data cannot be made publicly available; readers should contact the corresponding author for details.

## HIGHLIGHTS

- Land rights disputes between the Bedouins and the Israeli government have led to poor access to safely managed water and sanitation.
- We found that only 44% of households sampled in five Bedouin localities had access to both a safely managed water source and safely managed sanitation.
- In one unrecognized village, access to safely managed water and sanitation was worse than in many of the world’s poorest countries.
