# Supplementary material for: Racial and ethnic disparities in access to safe water and sanitation in high-income countries: a case study among the Arab-Bedouins of Southern Israel
Source: J Water Sanit Hyg Dev. Author manuscript; Available in PMC 2025 Mar 7. (PMC11887579; doi:10.2166/washdev.2023.162)
Supplement: Supplementary Material [file NIHMS2058729-supplement-Supplementary_Material.docx]

**Supplemental Materials:**

**Racial and ethnic disparities in access to safe water and sanitation in high-income countries: a case study among the Arab-Bedouins of Southern Israel**

Jesse D. Contreras^1^, Haneen Shibli^2,3^, Marisa C. Eisenberg^1^, Ahmad S. Muhammad^4^, Nadav Davidovitch^2^, Mark A. Katz^1,2^, Nihaya Daoud^2^, and Joseph N.S. Eisenberg^1^*

1: Department of Epidemiology, University of Michigan, Ann Arbor, MI, United States.

2: School of Public Health, Faculty of Health Sciences, Ben-Gurion University of the Negev, Be’er Sheva, Israel

3: PREPARED Center for Emergency Response Research, Israel

4: The Galilee Society for Health Research & Services, Rikaz Data Bank, Shefa-amer, Israel.

*Corresponding Author: 1415 Washington Heights, Ann Arbor, MI 48109-2029, United States, [jnse@umich.edu](mailto:jnse@umich.edu).

**Short Title:** Disparities in access to safe water and sanitation among the Bedouins of Israel

**Bedouin WASH Survey**

**INSTRUCTIONS**

Ask these questions to the *female head of household* or *primary caregiver* capable of answering for the household. Please read all options aloud unless otherwise noted.

| Num. | Question | | | Responses | | | | | Coding | |
| --- | --- | --- | --- | --- | --- | --- | --- | --- | --- | --- |
|  | ***Interviewer Questions*** | | | | | | | |  | |
| I1 | What is the name of the interviewer? | | | XXX ………………………………………………  XXX  XXX  XXX | | | | | 1  2  3  ... | |
| I2 | What is the identification number of the household? | | | [Enter 4-digit identification number]  ________________ | | | | |  | |
| I3 | In which village is the household located? | | | XXX  XXX  XXX  XXX | | | | | 1  2  3  ... | |
| I4 | Was an informed consent form signed by the participating woman? | | | Yes  No | | | | | 1  0 | |
|  | ***Household Demographics*** | | | | | | | |  | |
| D1 | What is your first name? | | | [Record name]  _________________ | | | | |  | |
| D2 | How long have you been living continuously in (NAME OF CITY, TOWN, OR VILLAGE OF RESIDENCE)? | | | [Record number of years:  Use 0 for less than 1 year  Use 95 for always  Use 96 for visitor]  ________________ years | | | | |  | |
| [D2 Skip Pattern: If 95 or 96 then go to D5, else go to D3] | | | | | | | | | | |
| D3 | Just before you moved here, did you live in a city, in a town, or in a rural area? | | | -City  -Town  -Rural area  -No response | | | | | 1  2  3  98 | |
| D4 | Just before you moved here, which region or type of rural area did you live in? | | | -Jerusalem district  -Northern district  -Haifa district  -Central district  -Tel Aviv district  -Southern district (Jewish cities other than Be’er Sheva)  -Be’er Sheva  -The West Bank  -Hevron  -Sinai  -Other Bedouin town (specify):___________  -Other place outside of Israel  -Don’t know/don’t remember | | | | | 1  2  3  4  5  6  7  8  9  10  11  12  13  98 | |
| D5 | Have you ever attended school? | | | -Yes  -No  -No response | | | | | 1  0  98 | |
| [D5 Skip Pattern: If 1 then go to D6, else go to D7] | | | | | | | | | | |
| D6 | What is the highest level of school you completed? | | | -Primary  -Secondary  -High school  -College or not academic education (e.g. teacher’s college)  -University education (BA, MA,Phd)  -Don’t know/don’t remember | | | | | 1  2  3  4  5  98 | |
| D7 | What is your marital status? | | | -Currently married  -Separated  -Divorced  -Widowed  -Never married  -No response | | | | | 1  2  3  4  0  98 | |
| [D8 Skip Pattern: If 1 then go to D9, else go to D15] | | | | | | | | | | |
| D8 | Does your (husband/partner) have other wives or does he live with other women as if married? | | | -Yes  -No  -Don’t know/no response | | | | | 1  0  98 | |
| D9 | Including yourself, in total, how many wives or live-in partners does he have? | | | [Record total number of wives and live-in partners]  ____________  -Don’t know/no response | | | | | 98 | |
| D10 | Are you the first, second, … wife? | | | [Record rank]  ___________  -Don’t know/no response | | | | | 98 | |
| D11 | Has your husband ever attended school? | | | -Yes  -No  -No response | | | | | 1  0  98 | |
| [D12 Skip Pattern: If 1 then go to D13, else go to D14] | | | | | | | | | | |
| D12 | What is the highest level of school your husband has completed? | | | -Primary  -Secondary  -High school  -College or not academnic education (e.g. teacher’s college)  -University education (BA, MA,Phd)  -Don’t know/don’t remember | | | | | 1  2  3  4  5  98 | |
| D13 | For this survey, a household includes all adults and children who live in the same housing unit or part of it, and share food or any other living arrangements.  How many people over 15 years old usually live in your household, including yourself? | | | # | Name | Gender (M/F) | Age (years) | |  | |
|  |  |  |  | 1. |  |  |  | |  |  |
|  |  |  |  | 2. |  |  |  | |  |  |
| D14 | How many people under 15 years old usually live in your household, including teenagers, children, infants, and newborns? | | | # | Name | Gender (M/F) | Age (years) | Is this your child? (Y/N) | Is this child currently attending school? (Y/N) | |
|  |  |  |  | 1. |  |  |  |  |  | |
|  |  |  |  | 2. |  |  |  |  |  | |
|  | ***Household Characteristics*** | | | | | | | |  | |
| H1 | What is the main source of drinking water for members of your household? | | | -Piped by government from public source to household/neighbor’s household  -Piped from public source to household/neighbor’s household, not by government  -Collected from public source, not piped to household/neighbor’s household  -Tube well or borehole  -Protected well  -Unprotected well  -Protected spring  -Unprotected spring  -Rainwater  -Tanker truck  -Cart with small tank  -Surface water (river/dam/lake/pond/canal/ irrigation channel)  -Bottled water  -Other (specify): __________________________ | | | | | 1  2  3  4  5  6  7  8  9  10  11  12  13  14 | |
| [H1 Skip Pattern: If 2, 3, 4, or 5 then go to H3, else go to H2] | | | | | | | | | | |
| H2 | Where is the main source of drinking water located? | | | -In own dwelling  -In own yard/plot  -In neighbor’s dwelling  -In neighbor’s yard/plot  -Other (specify): __________________________  -Don’t know | | | | | 1  2  3  4  5  98 | |
| H3 | What is the main source of water used for household purposes, such as cooking and handwashing? | | | -Piped by government from public source to household/neighbor’s household  -Piped from public source to household/neighbor’s household, not by government  -Collected from public source, not piped to household/neighbor’s household  -Tube well or borehole  -Protected well  -Unprotected well  -Protected spring  -Unprotected spring  -Rainwater  -Tanker truck  -Cart with small tank  -Surface water -(river/dam/lake/pond/canal/ irrigation channel)  -Bottled water  -Other (specify): __________________________ | | | | | 1  2  3  4  5  6  7  8  9  10  11  12  13  14 | |
| [H3 Skip Pattern: If 2, 3, 4, or 5 then go to H7, else go to H4] | | | | | | | | | | |
| H4 | Where is the main source of water located for household purposes? | | | -In own dwelling  -In own yard/plot  -In neighbor’s dwelling  -In neighbor’s yard/plot  -Other (specify): __________________________  -Don’t know | | | | | 1  2  3  4  5  98 | |
| [H5 Logic: Will only appear if 1 selected for H1 or H3] | | | | | | | | | | |
| H5 | How often does your household have access to the public water source? | | | -All the time  -Sometimes  -Very rarely  -Don’t know | | | | | 1  2  3  0  98 | |
| [H6 Logic: Will only appear if 1 selected for H1 or H3] | | | | | | | | | | |
| H6 | Does Makarot manage this drinking water source? | | | -Yes  -No  -Don’t know | | | | | 1  0  98 | |
| H7 | How do you usually travel to your main source of drinking water to collect water? | | | -Walk  -Drive own car  -Drive in tramp  -Ride in transit vehicle  -Ride an animal  -I do not travel to collect drinking water  -Other (specify): ________________  -Don’t know | | | | | 1  2  3  4  5  6  7  98 | |
| [H7 Skip Pattern: If 6 or 98 then go to H9, else go to H8] | | | | | | | | | | |
| H8 | How long does it take to travel from your household to your main source of drinking water? | | | [Record time in hours and minutes]  _______ hours . ________ minutes | | | | |  | |
| H9 | In the past week, was the water from your main source of drinking water not available for at least one full day? | | | -Yes  -No  -Don’t know/Don’t remember | | | | | 1  0  98 | |
| H10 | Do you do anything to the drinking water to make it safer to drink, such as boiling or using a filter? | | | -Yes  -No  -Don’t know | | | | | 1  0  98 | |
| [H10 Skip Pattern: If 1 then go to H11, else go to H12] | | | | | | | | | | |
| H11 | What do you usually do to make the drinking water safer to drink? | | | [Record all mentioned]  -Boil  -Add bleach/chlorine  -Strain through a cloth  -Use water filter (ceramic/sand/composite/etc.)  -Solar disinfection  -Let it stand and settle  -Other (specify): __________________________  -Don’t know | | | | | 1  2  3  4  5  6  7  98 | |
| H12 | What kind of toilet do members of your household usually use? | | | -Toilet that flushes  -Pour flush latrine  -Ventilated improved pit latrine  -Pit latrine with slab  -Pit latrine without slab/open pit  -Composting toilet  -Bucket toilet  -Hanging toilet/hanging latrine  -No facility/bush/field  -Other (specify): _________________________ | | | | | 1  2  3  4  5  6  7  8  9  10 | |
| H13 | Do you share this toilet facility with other households? | | | -Yes  -No  -Don’t know | | | | | 1  0  98 | |
| [H13 Skip Pattern: If 1 then go to H14, else go to H15] | | | | | | | | | | |
| H14 | Including your own household, how many households use this facility? | | | [Record number of households]  _____________ households  -Don’t know | | | | | 98 | |
| H15 | Where is this toilet facility located? | | | -In own dwelling  -In own yard/plot  -Elsewhere  -Don’t know | | | | | 1  2  3  98 | |
| H16 | Is the toilet facility connected to a sewage system? | | | -Yes  -No  -Don’t know | | | | | 1  0  98 | |
| H17 | Where does the toilet flush to? | | | -Piped sewer system  -Septic tank  -Pit latrine  -Cesspit  -Discharge directly to environment  -Connect to pipe, then environment  -Other (specify): _____________________  -Don’t know | | | | | 1  2  3  4  5  6  7  98 | |
| [H18 Logic: Will only appear if 2 or 4 selected for H17] | | | | | | | | | | |
| H18 | Do you share this cesspit or septic tank with other households? | | | -Yes  -No  -Don’t know | | | | | 1  0  98 | |
| [H18 Skip Pattern: If 1 then go to H19, else go to H20] | | | | | | | | | | |
| H19 | Including your own household, how many households use this cesspit or septic tank? | | | [Record number of households]  _______________ households  -Don’t know | | | | | 98 | |
| H20 | Do you have a separate room in this household that is used as a kitchen? | | | -Yes  -No  -Don’t know | | | | | 1  0  98 | |
| H21 | How many rooms in this household are used for sleeping? | | | -1  -2  -3  -4  -5  -6  -7 or more | | | | | 1  2  3  4  5  6  7 | |
| H22 | Does this household own any livestock, herds, other farm animals, or poultry? | | | -Yes  -No  -Don’t know | | | | | 1  0  98 | |
| [H22 Skip Pattern: If 1 then go to H23, else go to H25] | | | | | | | | | | |
| H23 | Which of the following animals does this household own? | | | [Select all that apply]  -Milk cows, or bulls  -Horses, donkeys, or mules  -Goats  -Sheep  -Chickens, or other poultry  -Camels | | | | | 1  2  3  4  5  6 | |
| H24 | How are the animals kept? | | | [Select all that apply]  -Roam free  -Fenced in near household  -Fenced in elsewhere  -In animal shed  -Tied up  -In neighbor’s yard/plot  -Other (specify): _____________________  -Don’t know | | | | | 1  2  3  4  5  6  7  98 | |
| H25 | Does any member of this household own any agricultural land? | | | -Yes  -No  -Don’t know | | | | | 1  0  98 | |
| [H25 Skip Pattern: If 1 then go to H26, else go to H27] | | | | | | | | | | |
| H26 | What are all the sources of water used by your household for agricultural irrigation? | | | -Piped by government from public source to household/neighbor’s household  -Piped from public source to household/neighbor’s household, not by government  -Collected from public source, not piped to household/neighbor’s household-Tube well or borehole  -Protected well  -Unprotected well  -Protected spring  -Unprotected spring  -Rainwater  -Tanker truck  -Cart with small tank  -Surface water (river/dam/lake/pond/canal/ irrigation channel)  -Reused water from the household  -Other (specify): __________________________ | | | | | 1  2  3  4  5  6  7  8  9  10  11  12  13  14 | |
| H27 | How does your household mainly dispose of garbage? | | | [Select all that apply]  -Dumped into street/yard  -Dumped into wadi  -Burned  -Fed to animals  -Taken to landfill  -Collected by government services  -Collected by non-government services  -Don’t know | | | | | 1  2  3  4  5  6  7  98 | |
| [H27 Skip Pattern: If 6 selected then go to H28, else go to H29] | | | | | | | | | | |
| H28 | How often does the government collect garbage from your household? | | | -Once a week  -Twice a week  -Once every two weeks  -Less than once every two weeks  -Don’t know | | | | | 1  2  3  4  98 | |
| H29 | Does your household have: | | | [Read each response aloud  Select all that apply]  Electricity  A radio  A television  A color television  A video/DVD player  A non-mobile phone  Access to the internet  A refrigerator  An air conditioner  A freezer  A water heater  A dishwater  An automatic washing machine  Other washing machine  A sewing machine  An electric fan  A satellite dish  A generator  Solar panels  A water cooler  A microwave  A computer  A bed or sofa  A mobile-phone  A smart phone | | | | | 1  2  3  4  5  6  7  8  9  10  11  12  13  14  15  16  17  18  19  20  21  22  23  24  25 | |
|  | ***Individual WASH Behaviors*** | | | | | | | |  | |
| IW1 | The last time you defecated, did you use a toilet/latrine? | | | -Yes  -No  -Don’t remember/no response | | | | | 1  0  98 | |
| IW2 | The last time you defecated at home, did you use a toilet/latrine? | | | -Yes  -No  -Don’t remember/no response | | | | | 1  0  98 | |
| IW3 | In the last week, when you defecated at home, how often did you use your household toilet/latrine? | | | -Never  -Rarely  -Sometimes  -Most of the time  -Always  -Don’t know/no response | | | | | 0  1  2  3  4  98 | |
| IW4 | Are you employed, or do you do any work other than your housework, such as working in the family business or working on the family farm? | | | -Yes  -No  -Don’t know/No response | | | | | 1  0  98 | |
| [IW4 Skip Pattern: If 1 then go to IW5, else go to next section (HF)] | | | | | | | | | | |
| IW5 | The last time you defecated at work, did you use a toilet/latrine? | | | -Yes  -No  -Don’t know/no response | | | | | 1  0  98 | |
| IW6 | In the last week, when you defecated at work, how often did you use a toilet/latrine? | | | -Never  -Rarely  -Sometimes  -Most of the time  -Always  -Don’t know/no response | | | | | 0  1  2  3  4  98 | |
|  | ***Household Finances*** | | | | | | | |  | |
| [HF1 Logic: Will only appear if 1 selected for IW4] | | | | | | | | | | |
| HF1 | What is your occupation? That is, what kind of work do you mainly do? | | | [Record response]  ________________ | | | | |  | |
| HF2 | In which city do you work? | | | [Record response]  _____________________ | | | | |  | |
| [HF2 Logic: Will only appear if 1 selected for D8] | | | | | | | | | | |
| HF3 | What is your husband/partner’s occupation? That is, what kind of work does he mainly do? | | | [Record respond]  __________________ | | | | |  | |
| HF4 | In which city does your husband work? | | | [Record response]  _____________________ | | | | |  | |
| HF5 | Does your household own this or any other house? | | | -Yes  -No  -Don’t know/no response | | | | | 1  0  98 | |
| HF6 | What are the current sources of income for you and/or your spouse? | | | -Work  -Benefit from National Insurance (unemployment, disability, child allowance)  -Income from land or property  -Other (specify): ______________________  -Don’t know/no response | | | | | 1  2  3  4  98 | |
|  | ***Healthcare Access*** | | | | | | | |  | |
| HA1 | Which health fund do you belong to? | | | -Clalit Health Services  -Leumit Health Fund  -Maccabi Healthcare Services  -Meuhedet Health Fund  -Don’t know | | | | | 1  2  3  4  98 | |
| HA2 | If you were having a health problem, where would you seek advice or treatment? | | | -Primary Care Clinic (Kupah) in village  -Primary Care Clinic (Kupah) in a nearby village or city  Private physician’s clinic  -Terem Clinic (emergency clinic)  -Soroka Hospital in Be’er Sheva  -Traditional healer/herbalist  -Alhayat private ER clinic  -Would not seek advice or treatment  -Other (specify): _____________________  -Don’t know/no response | | | | | 1  2  3  4  5  6  7  8  9  8  98 | |
| [HA2 Skip Pattern: If 8 or 98 then go to HA5, else go to HA3] | | | | | | | | | | |
| HA3 | What mode of transportation would you take to get to the clinic/hospital that you mentioned above? | | | -Private car/truck  -Taxi  -Bus  -Walking  -No response | | | | | 1  2  3  4  98 | |
| [HA3 Skip Pattern: If 98 then go to HA5, else go to HA4] | | | | | | | | | | |
| HA4 | How much time does it take to get to the clinic/hospital that you mentioned above? | | | [Record time in minutes]  ____________________  -Don’t know | | | | | 98 | |
| HA5 | Did you consult a doctor the last time you were sick? | | | -Yes  -No  -Don’t know/no response | | | | | 1  0  98 | |
| [HA5 Skip Pattern: If 0 then go to HA6, else go to HA7] | | | | | | | | | | |
| HA6 | | Why didn’t you consult a doctor the last time you had a health problem? | | -The doctor’s office was too far away/difficult to get to  -Lack of money  -Lack of transport  -Used traditional medicine  -Self-medication or referral to pharmacy  -The illness wasn’t very serious and I didn’t think I needed a doctor  -Other (specify): _________________  -Don’t know/no response | | | | | 1  2  3  4  5  6  7  8  98 | |
| HA7 | | Did you consult a doctor the last time one of your children was sick? | | -Yes  -No  -Don’t know/no response | | | | | 1  0  98 | |
| [HA7 Skip Pattern: If 0 then go to HA8, else go to HA9] | | | | | | | | | | |
| HA8 | | Why didn’t you consult a doctor the last time your child was sick? | | -The doctor’s office was too far away/difficult to get to  -Lack of money  -Lack of transport  -Used traditional medicine  -Self-medication or referral to pharmacy  -The illness wasn’t very serious and I didn’t think I needed a doctor  -Other (specify): _________________  -Don’t know/no response | | | | | 1  2  3  4  5  6  7  8  98 | |
| HA9 | | Can you visit health provider alone or is someone required to accompany you? | | -Can go by self  -Required to be accompanied  -Don’t know/no response | | | | | 1  2  98 | |
| HA10 | | Do you have a primary care physician? | | -Yes  -No  -Don’t know/no response | | | | | 1  0  98 | |
| [HA10 Skip Pattern: If 1 then go to HA11, else go to next section (CH)] | | | | | | | | | | |
| HA11 | | Are you able to communicate with your primary care physician in a language that you are comfortable with? | | -Yes  -No  -Don’t know/no response | | | | | 1  0  98 | |
| HA12 | | Do you feel that your primary care physician understands your culture? | | -Strongly agree  -Agree  -Neutral  -Disagree  -Strongly disagree  -Don’t know | | | | | 1  2  3  4  5  98 | |
|  | | ***Child Health and Nutrition*** | | | | | | |  | |
| CH1 | | [READ ALOUD]  As a reminder, for this survey, a household includes all adults and children who live in the same housing unit or part of it, and share food or any other living arrangements.  How many children under five years old are part of this household? | | -0  -1  -2  -3  -4  -5+ | | | | | 0  1  2  3  4  5 | |
| [CH1 Skip Pattern: If 0 then go to Travel Patterns Section, else go to CH2] | | | | | | | | | | |
| CH2 | | Are there children under five years old in this houshold, that are not your own, that you take care of? | | -Yes  -No  -No Response | | | | | 1  0  98 | |
| [CH2 Skip Pattern: If 1 then go to CH3, else go to Child Health Table] | | | | | | | | | | |
| CH3 | | How many children under five years old in this household, that are not your own, do you usually take care of? | | -1  -2  -3  -4  -5+ | | | | | 1  2  3  4  5 | |
| [Child Health Table Logic: will only show if CH1 does not equal 0 or CH2 equals 1] | | | | | | | | | | |
|  | | ***Child Health Table*** | | | | | | |  | |
| CH4 | | Name | | _________________ | | | | |  | |
| CH5 | | Date of Birth | | ____-______-________ [DD/MM/YY] | | | | |  | |
| CH6 | | Is this your child? | | -Yes  -No | | | | | 1  0 | |
| CH7 | | Is (NAME) still being breastfed? | | -Yes  -No  -No response | | | | | 1  0  98 | |
| CH8 | | Has (NAME) had three or more loose or watery stools, or more loose or watery stools than usual, one or more days in the last week? | | -Yes  -No  -Don’t know | | | | | 1  0  98 | |
| [CH8 Skip Pattern: If 1 then go to CH9, else go to CH15] | | | | | | | | | | |
| CH9 | | In the last week, how many days has (NAME) had loose or watery stools? | | -1  -2  -3  -4  -5  -6  -7  -Don’t know | | | | | 1  2  3  4  5  6  7  98 | |
| CH10 | | When (NAME) had diarrhea, was (NAME) given less than usual to drink, about the same amount, or more than usual to drink? | | -Much less  -Somewhat less  -About the same  -More  -Nothing to drink  -Don’t know | | | | | 1  2  3  4  0  98 | |
| CH11 | | When (NAME) had diarrhea, was (NAME) given less than usual to eat, about the same amount, more than usual, or nothing to eat? | | -Much less  -Somewhat less  -About the same  -More  -Stopped food  -Don’t know | | | | | 1  2  3  4  5  98 | |
| CH12 | | Did you seek advice or treatment for the diarrhea from any source? | | -Yes  -No  -Don’t know/no response | | | | | 1  0  98 | |
| [CH12 Skip Pattern: If 1 then go to CH13, else go to CH15] | | | | | | | | | | |
| CH13 | | Where did you seek advice or treatment? | | [Select all that apply]  -Government hospital  -Government health center  -Government health post  -Public mobile clinic  -Public fieldworker  -Other public sector (specify): ____________  -Private hospital/clinic  -Pharmacy  -Private doctor  -Private mobile clinic  -Private fieldworker  -Other private medical sector (specify): ____  -Shop  -Traditional practitioner/herbalist  -Market  -Itinerant drug seller  -Other (specify): ________________  -Don’t know/no response | | | | | 1  2  3  4  5  6  7  8  9  10  11  12  13  14  15  16  17  98 | |
| CH14 | | Was (NAME) given any of the following at any time since (NAME) started having the diarrhea? | | [Select all that apply]  -A fluid made from a special packet called “Electrorice” or “Tiptipot Mineral”  -A pre-packaged ORS liquid  -A government-recommended homemade fluid  -Zinc tablets or syrup  -Antibiotic pill or syrup  -Antimotility pill or syrup  -Other pill or syrup (not antibiotic or antimotility)  -Antibiotic injection  -Atimotility injection  -Unknown injection  -(IV) Intravenous fluid  -Home remedy/herbal medicine  -Other (specify): ____________________  -Don’t know/no response | | | | | 1  2  3  4  5  6  7  8  9  10  11  12  13  98 | |
| CH15 | | Has (NAME) been ill with fever at any time in the last week? | | -Yes  -No  -Don’t know/no response | | | | | 1  0  98 | |
| [CH15 Skip Pattern: If 1 then go to CH16, else go to CH18] | | | | | | | | | | |
| CH16 | | At any time during the illness, did (NAME) take any drugs for the illness? | | -Yes  -No  -Don’t know/no response | | | | | 1  0  98 | |
| [CH16 Skip Pattern: If 1 then go to CH17, else go to CH18] | | | | | | | | | | |
| CH17 | | What drugs did (NAME) take? | | [Select all that apply]  -Antibiotic pill/syrup  -Antibiotic injection/IV  -Aspirin  -Acetaminophen  -Ibuprofen  -Other (specify): ________________ | | | | | 1  2  3  4  5  98 | |
| CH18 | | In the last 7 days, was (NAME) given: | | [Select all that apply]  -Multiple micronutrient food, like “Sprinkles”  -Therapeutic foods, like Ensure, Glucerna, Naphrokar, or Jabitti  -Ready to use supplemental food | | | | | 1  2  3 | |
| ***Continuation of Child Health Table*** | | | | | | | | | | |
|  | | | ***Immunizations*** | | | | | | |  |
| IM1 | | | Do you have a vaccination booklet (Pinkas Chisunim) or other document where (NAME)’s vaccinations are written down? | -Yes, has only a booklet  -Yes, has only another document  -Yes, has booklet and other document  -No, no booklet and no other document | | | | | | 1  2  3  0 |
| [IM2 Logic: Will only appear if 2 or 0 selected for IM1] | | | | | | | | | | |
| IM2 | | | Did you ever have a vaccination booklet (Pinkas Chisunim) for (NAME)? | -Yes  -No  -Don’t know | | | | | | 1  0  98 |
| [IM3 Logic: Will only appear if 1, 2, or 3 selected for IM1] | | | | | | | | | | |
| IM3 | | | May I see the vaccination booklet (Pinkas Chisunim) where (NAME)’s vaccinations are written down? | -Yes, only booklet seen  -Yes, only other document seen  -Yes, booklet and other document seen  -No booklet and no other document seen | | | | | | 1  2  3  0 |
| [IM3 Skip Pattern: If 0 then go to IM5, else go to IM4] | | | | | | | | | | |
| IM4 | | | RECORD IF FOLLOW VACCINATIONS WERE RECEIVED FROM THE BOOKLET/DOCUMENT | [Select all that apply]  -Inactivated polio virus (IPV) 1  -Inactiviated polio virus (IPV) 2  -Inactivated polio virus (IPV) 3  -Oral polio virus (bOPV) 1  -Oral polio virus (bOPV) 2  -Rotavirus 1  -Rotavirus 2 | | | | | | 1  2  3  4  5  6  7 |
| [IM5 Logic: Ask if any of the options 1-7 in IM4 are not selected] | | | | | | | | | | |
| IM5 | | | In addition to what is recorded on (this document/these documents), did (NAME) receive these other vaccinations, including vaccinations received in campaigns or immunization days or child health days? | [Select all that apply]  -Inactivated polio virus (IPV) 1  -Inactiviated polio virus (IPV) 2  -Inactivated polio virus (IPV) 3  -Oral polio virus (bOPV) 1  -Oral polio virus (bOPV) 2  -Rotavirus 1  -Rotavirus 2 | | | | | | 1  2  3  4  5  6  7 |
| [Child Health Table Logic: Repeat table if there is another child under 5 years old]  Select one of the following:  **[Proceed to Child WASH Section]** OR **[Add another child under 5 table]** | | | | | | | | | | |
|  | | | ***Child WASH*** | | | | | | |  |
| CW1 | | | How many children under five in the household use diapers? | -None  -1  -2  -3  -4  -5  -All children under five use diapers  -Don’t know | | | | | | 0  1  2  3  4  5  77  98 |
| [CW1 Skip Pattern: If 0 or 98 then go to CW5, else go to CW2] | | | | | | | | | | |
| CW2 | | | What do you do with the diapers after they are used? | -Dispose in toilet or latrine  -Dispose of in open area  -Dispose of in a ditch/ravine/hole  -Dispose of in the garbage  -Bury them  -Burn them  -Wash and reuse cloth diaper  -Don’t know | | | | | | 1  2  3  4  5  6  7  98 |
| CW3 | | | Is there a specific location where children’s diapers are changed? | -Yes  -No  -Don’t know | | | | | | 1  0  98 |
| CW4 | | | Is the surface of the changing station cleaned after each time a diaper is changed? | -Yes  -No  -Don’t know | | | | | | 1  0  98 |
| [CW5 Logic: Do not ask if 77 chosen for CW1] | | | | | | | | | | |
| CW5 | | | For children not in diapers, what is usually done to dispose of stool? | -Used toilet/latrine  -Put/rinsed into toilet/latrine  -Put/rinsed into drain/ditch  -Thrown into garbage  -Buried  -Left in the open  -Other (specify): ____________________  -Don’t know | | | | | | 1  2  3  4  5  6  8  98 |
| CW6 | | | In general, do you wash the hands of children under five or do they wash their hands themselves? | -I wash them  -They wash themselves  -Both  -They are not washed  -Don’t know | | | | | | 1  2  3  4  98 |
|  | | | ***Travel Patterns*** | | | | | | |  |
|  | | | [READ ALOUD]  We now want to understand how people in this village travel inside and outside of Israel because travel between places can sometimes spread disease. | | | | | | |  |
| T1 | | | During the past week, have you traveled outside the village? | -Yes  -No  -Don’t know/no response | | | | | | 1  0  98 |
| [T1 Skip Pattern: If 1 then go to T2, else go to T3] | | | | | | | | | | |
| T2 | | | Where did you go? | [Select all that apply]  -Jerusalem district  -Northern district  -Haifa district  -Central district  -Tel Aviv district  -Southern district (Jewish cities other than Be’er Sheva)  -Be’er Sheva  -The West Bank  -Hevron  -Sinai  -Other Bedouin town (specify):___________  -Other place outside of Israel  -Don’t know/don’t remember | | | | | | 1  2  3  4  5  6  7  8  9  10  11  12  13  98 |
| [T3 Logic: Will only appear if 1 selected for D8] | | | | | | | | | | |
| T3 | | | During the past week, has your (husband/partner) traveled outside of the village? | -Yes  -No  -Don’t know/no response | | | | | | 1  0  98 |
| [T3 Skip Pattern: If 1 then go to T4, else go to T5] | | | | | | | | | | |
| T4 | | | Where did he go? | [Select all that apply]  -Jerusalem district  -Northern district  -Haifa district  -Central district  -Tel Aviv district  -Southern district (Jewish cities other than Be’er Sheva)  -Be’er Sheva  -The West Bank  -Hevron  -Sinai  -Other Bedouin town (specify):___________  -Other place outside of Israel  -Don’t know/don’t remember | | | | | | 1  2  3  4  5  6  7  8  9  10  11  12  13  98 |
| T5 | | | In the past week, have you had any visitors to your household? | -Yes  -No  -Don’t know | | | | | | 1  0  98 |
| [T5 Skip Pattern: If 1 then go to T6, else go to T7] | | | | | | | | | | |
| T6 | | | Where did your visitors come from? | [Select all that apply]  -Jerusalem district  -Northern district  -Haifa district  -Central district  -Tel Aviv district  -Southern district (Jewish cities other than Be’er Sheva)  -Be’er Sheva  -The West Bank  -Hevron  -Sinai  -Other Bedouin town (specify):___________  -Other place outside of Israel  -Don’t know/don’t remember | | | | | | 1  2  3  4  5  6  7  8  9  10  11  12  13  98 |
| [T7 Logic: Will only appear if children inputed in D16 table] | | | | | | | | | | |
| T7 | | | In what city, town, or village do the children in the household go to school? | [Record location of school]  ___________________________ | | | | | |  |
| [T8 Logic: Will only appear if children inputed in D16 table] | | | | | | | | | | |
| T8 | | | During the past week, other than going to school, have any of the children in the household traveled outside the village? | -Yes  -No  -Don’t know | | | | | | 1  0  98 |
| [T8 Skip Pattern: If 1 then go to T9, else go to end of survey] | | | | | | | | | | |
| T9 | | | Where did they go? | [Select all that apply]  -Jerusalem district  -Northern district  -Haifa district  -Central district  -Tel Aviv district  -Southern district (Jewish cities other than Be’er Sheva)  -Be’er Sheva  -The West Bank  -Hevron  -Sinai  -Other Bedouin town (specify):___________  -Other place outside of Israel  -Don’t know/don’t remember | | | | | | 1  2  3  4  5  6  7  8  9  10  11  12  13  98 |

**END OF SURVEY**

|  | ***Observations*** | |  |
| --- | --- | --- | --- |
|  | [READ ALOUD]  We would like to learn about the places that households use to wash their hands. | |  |
| O1 | Can you please show me where members of your household most often wash their hands? | -Observed, fixed place  -Observed, mobile  -Not observed, not in dwelling/yard/plot  -Not observed, no permission to see  -Not observed, other reason | 1  2  3  4  5 |
| [O1 Skip Pattern: If 3, 4, or 5 selected then go to O4, else go to O2] | | | |
| O2 | OBSERVE PRESENCE OF WATER AT THE PLACE FOR HANDWASHING. RECORD OBSERVATION. | -Water is available  -Water is not available  -Not observed | 1  0  98 |
| O3 | OBSERVE PRESENCE OF SOAP, DETERGENT, OR OTHER CLEANSING AGENT AT THE PLACE FOR HANDWASHING. RECORD OBSERVATION. | -Soap or detergent (bar/liquid/powder/paste)  -Ash, mud, sand  -None  -Not observed | 1  2  3  98 |
| O4 | OBSERVE MAIN MATERIAL OF THE FLOOR OF THE DWELLING. RECORD OBSERVATION. | -Earth/sand  -Dung  -Wood planks  -Palm/bamboo  -Parquet or polished wood  -Vinyl or asphalt strips  -Ceramic tiles  -Cement  -Carpet  -Other (specify): __________________  -Not observed | 1  2  3  4  5  6  7  8  9  10  98 |
| O5 | OBSERVE MAIN MATERIAL OF THE ROOF OF THE DWELLING. RECORD OBSERVATION. | -No roof  -Thatch/palm leaf  -Sod  -Rustic mat  -Palm/bamboo  -Wood planks  -Cardboard  -Metal  -Wood  -Calamine/cement fiber  -Ceramic tiles  -Cement  -Roofing shingles  -Other (specify): ____________________  -Not observed | 1  2  3  4  5  6  7  8  9  10  11  12  13  14  98 |
| O6 | OBSERVE MAIN MATERIAL OF THE EXTERIOR WALLS OF THE DWELLING. RECORD OBSERVATION. | -No walls  -Cane/palm/trunks  -Dirt  -Bamboo with mud  -Stone with mud  -Uncovered adobe  -Plywood  -Cardboard  -Reused wood  -Cement  -Stone with lime/cement  -Bricks  -Cement blocks  -Covered adobe  -Wood planks/shingles  -Other (specify): _____________________  -Not observed | 1  2  3  4  5  6  7  8  9  10  11  12  13  14  15  16  98 |
|  | [READ ALOUD]  We would like to learn about the types of latrines/toilets households have. | |  |
| O7 | Can you please show me this households’ toilet/latrine? | -Allowed to observe  -Not allowed to observe | 1  0 |
| [O7 Skip Pattern: If 1 then go to O8, else end observations] | | | |
| O8 | OBSERVE TOILET/LATRINE.  IF TOILET/LATRINE IS LOCATED OUTSIDE OF THE HOUSEHOLD: HAS THE PATH TO THE LATRINE BEEN WALKED? RECORD OBSERVATION. | -Yes  -No  -Latrine located inside  -Don’t know | 1  0  2  98 |
| O9 | OBSERVE TOILET/LATRINE.  IS THERE EVIDENCE OF USED ANAL CLEANSING MATERIAL? RECORD OBSERVATION. | -Yes  -No  -Don’t know | 1  0  98 |
| O10 | IS FECES DETECTABLE IN THE PIT? RECORD OBSERVATION. | -Yes  -No  -Flush latrine/toilet  -Don’t know | 1  0  2  98 |
| O11 | HOLE COVER/LID (IF CLEARLY PART OF THE ORIGINAL FACILITY) | -No hole cover present  -Hole cover defective, broken, or not used  -Hold cover placed over hole and tight fitting  -Not observed | 1  2  3  98 |
| O12 | DISPOSAL OF USED ANAL CLEANSING MATERIAL | -Soiled anal cleansing material accumulated on floor of latrine  -Some soiled anal cleansing material on latrine floor  -Used soiled anal cleansing material disposed of in pit/waste basket  -No soiled anal cleansing material visible  -Not observed | 1  2  3  4  98 |
| O13 | [IF FLUSH LATRINE/TOILET]  BOWL WATER SEAL. RECORD OBSERVATION. | -Water seal not maintained in bowl and bowl very dirty with solid or smeared feces  -Water seal is present but bowl contains fecal material, anal cleansing material, other materials  -Water seal is maintained in bowl and bowl is free of other contents: fecal matter, smeared feces, used anal cleansing material  -Not flush latrine/toilet  -Not observed | 1  2  3  4  98 |
| O14 | [IF FLUSH LATRINE/TOILET]  BOWL FLUSHING | -Water for flushing not present or easily accessible  -Water for flushing not present but accessible nearby : e.g. within 20 meters of toilet  -Water for flushing present in the bathroom: piped or in bucket/reservoir  -Not flush latrine/toilet  -Not observed | 1  2  3  4  98 |
| O15 | FLOOR OF BATHROOM/LATRINE | -Abundant dried and/or fresh fecal matter, urine, and/or used anal cleansing material on floor  -Some fecal matter or smeared feces, urine, and/or anal cleansing material on floor  -Very little or no fecal matter, smeared feces, urine, or anal cleansing material on floor  -Not observed | 1  2  3  98 |
| O16 | RECEPTACLE FOR ANAL CLEANSING MATERIAL | -No receptacle for anal cleansing material present  -Receptacle for anal cleansing material present but unused  -Receptacle for anal cleansing material present and used  -Not observed | 1  2  3  98 |
| IC1 | RECORD ANY COMMENTS RELATED TO THIS INTERVIEW | __________________________________________  __________________________________________  __________________________________________ |  |
